# Patient and Family Engagement During Challenging Times: What Works and What Does Not?

**DOI:** 10.7759/cureus.14814

**Published:** 2021-05-03

**Authors:** Muhammad Hasan Abid, Muhammad Mohsin Abid, Ruqayya Shahid, Jamal Al Nofeye, Iqbal Ratnani

**Affiliations:** 1 Internal Medicine, Institute for Healthcare Improvement, Boston, USA; 2 Internal Medicine, Jinnah Medical College, Peshawar, PAK; 3 Dentistry, Fatima Memorial Hospital College of Medicine and Dentistry, Lahore, PAK; 4 Oncology, Armed Forces Hospitals, Taif, SAU; 5 Critical Care Medicine, Debakey Heart and Vascular Center, Houston, USA

**Keywords:** patient experience, patient satisfaction, physician-patient relations, communication, patient reported outcome measures, patient-centered care, covid-19, sars-cov-2

## Abstract

A paradigm shift towards enhanced strategies to effectively engage patients and families in delivering safe and high-quality healthcare services was observed during recent times, particularly in the last decade. Immediately prior to the coronavirus disease 2019 (Covid-19) pandemic, the tri-institutional global healthcare quality reports from the National Academies of Sciences, Engineering, and Medicine, World Bank Group, and Lancet Global Health Commission reported the patient and family engagement measures used globally, highlighting the variations across the regions of the world. Through a pandemic for more than a year now, we aim to present the key lessons learned from practices and strategies to proactively engage patients and families. These strategies may continue to be implemented in the post-Covid-19 pandemic era to improve patient and family-centered care.

## Introduction and background

Person-centered care is the cornerstone of high-quality healthcare service as outlined by the Institute of Medicine (now National Academy of Medicine) in its 2001 seminal report, Crossing the Quality Chasm [1]. Delivering person-centered care requires “providing care that is respectful of and responsive to individual patient preferences, needs, and values and ensuring that (these) values guide all clinical decisions” [1]. Globally the patient-centered care measurement indicators demonstrate huge variations, as mentioned in the following tri-institutional reports published during late 2018. In its report “Crossing the Global Quality Chasm: Improving the Healthcare Worldwide,” the National Academies of Sciences, Engineering, and Medicine describes patient-centered care as the levels of dissatisfaction for overall care (2.2% to 54.3% globally) [2]. The “Lancet Global Health Commission High-Quality Health Systems in the Sustainable Development Goals Era: Time for a Revolution” reports the patient-centered care measurement as poor user experience upon contact with the healthcare systems (34% of people in low-income and middle-income countries) [3]. The “World Health Organization, Organization for Economic Co-operation and Development, and The World Bank Delivering Quality Health Services: A Global Imperative” report mentions the patient-centered care measured as doctors providing easy-to-understand explanations (age-standardized rate of 81.3 per 100 patients for high-income countries in the Organization for Economic Co-operation and Development) [4]. These reports called upon the healthcare stakeholders across the globe to work collaboratively on reducing the ambiguity on what it actually means or how to measure patient-centeredness through a standardized method best.

In the midst of this ongoing global healthcare paradigm shift towards patient and family-centered care, the emergence of the severe acute respiratory syndrome coronavirus 2 (SARS-COV-2) in December 2019 led to a novel coronavirus disease 2019 (Covid-19) pandemic, catalyzing a chain of rapid worldwide transformations in the practices of delivering healthcare services [5,6]. The contagion spread through the communities globally, overwhelming healthcare system capacity [6]. Governments and healthcare stakeholders locally, regionally, and internationally rushed to implement multiple measures to prevent disease transmission [7,8]. As a result, patients faced significant care disruptions of healthcare services. Globally, healthcare systems faced a dual challenge to work on innovative approaches to deliver healthcare during the Covid-19 pandemic and at the same time provide continued care to the non-Covid-19 patients [9-11]. We aim to present various lessons learned globally on patient and family engagement, focusing on the approaches that can continue to prove effective in the post-Covid-19 pandemic era.

## Review

Key lessons learned globally for strategic implementation and continued use in healthcare to improve patient-centered care:

*1.* *Co-production of Healthcare Services as Core Strategy for Patient and Family Engagement*

Healthcare service outcomes (good and bad) are co-produced or co-created by healthcare professionals in relation to one another and people seeking help to restore or maintain health for themselves and their families [12,13]. In its functional essence, ‘co-production' means that we have patients onboard the design team co-creating from the beginning of a healthcare service improvement effort to achieve high quality, value-based, low cost, and sustainable outcomes with a shared vision [14]. It is the equal partnership between healthcare providers bringing the wealth of ‘learned experience’ and the patients having the relevant ‘lived experience’ in a healthcare setting that provides a whole story demonstrating what matters most to the patients [15]. Only then the healthcare organizations can co-design and co-produce healthcare improvement through robust, innovative, and practical experience-based change ideas [15]. The Covid-19 pandemic underscores the importance of patient-and-family-centeredness contributing to a loved one’s quality of care [8]. An international multi-stakeholder effort led by US-based Planetree International developed “Person-Centered Guidelines for Preserving Family Presence in Challenging Times” to globally encourage the frontline healthcare staff and organizations in delivering patient-and-family-centered care during the Covid-19 pandemic [8]. Such multi-stakeholder global-level initiatives are of high importance and should continue in the post-Covid-19 era to minimize the variation in the patient-and-family-centeredness measures across the healthcare systems around the world.

Moreover, reducing patient and family engagement barriers to effectively co-produce in a rapid turnaround time is also of utmost importance [14]. An ‘Always Event’ (refers to aspects of the patient experience that are so important to patients and families that healthcare providers must perform them consistently for every patient, every time) approach has been used by more than 50% of healthcare providers in England with an aim to activate patients and providers to co-produce and commit to change [14,15]. Recently, National Steering Committee for Patient Safety released the “Safer Together: A National Action Plan to Advance Patient Safety,” highlighting the total system approach to advance patient safety through healthcare organizations committed to the goal of fully engaging patients, families, and care partners in all aspects of care across all levels using key tactics like ‘AHRQ Questions Are the Answers,’ ‘Ask Me 3,’ ‘Choosing Wisely,’ ‘Prepare for Your Care,’ ‘The Conversation Project,’ ‘Cake,’ ‘What Matters to You,’ ‘Open Notes,’ ‘Patient and Family Advisory Councils for Quality and Safety,’ and ‘Patient and Family Advisor Program’ [16-18]. These co-production and co-design playbook techniques can be used worldwide in a standardized manner to improve patient safety and quality of care, ultimately advancing patient-and-family-centered care.

2. Leverage Technological Advancements to Enhance Patient and Family Engagement

The recent challenging times in the context of the Covid-19 pandemic have highlighted the continued importance of solid human relationships, leading to telehealth’s rapid growth to minimize interruption of vital clinical services [11,19]. A survey of US physicians in 2020 revealed that about half of the physicians are treating patients using virtual visits [20]. Telehealth solutions have also been used extensively during the Covid-19 pandemic in the inpatient settings to promote patient and family engagement [21]. Telehealth provides a real opportunity for the future of healthcare delivery. However, there are few hurdles in telehealth’s path to a sustainable healthcare delivery model. Firstly, the lack of a global framework to license healthcare workers and protect patient data privacy allowing cross-state or cross-country telehealth consultations [19,22]. Secondly, an area of increasing concern is to ensure that telehealth services are equitable and accessible by all the communities, patient groups, and ethnicities [22,23]. Finally, the telehealth services’ payment structure needs to be devised. This can be radically different across jurisdictions [7,19,22]. In order to increase the use of telehealth services in the post-pandemic era with the recovery of the normal daily processes or ‘new normal,’ the healthcare organizations, providers, and frontline staffs need to embrace telehealth technologies using data-driven guidelines of the patient-and-family-centered care for a sustainable and scalable implementation of telehealth into the routine clinical care [7,22].

Artificial Intelligence (AI), Machine Learning (ML), and Natural Language Processing (NLP) in the healthcare industry have been gaining popularity recently to solve the healthcare delivery operational issues mainly due to its capacity to analyze complex big data sets for accurate risk prediction, needs forecasting, and improving population-based healthcare outcomes [24]. One such protocol for ML algorithms can utilize patient data to predict the risk of poor patient and family engagement [25]. Such methodological approaches will help alarm the healthcare providers about the patients at risk of poor patient experience and low patient and family engagement to utilize more enhanced and focused strategies when dealing with such patients and families [25]. AI, ML, and NLP provide an opportunity for healthcare teams to collaborate while designing the healthcare technological solutions or tools that are capable of offloading the provider tasks like patient record review, provider order entry, and clinical documentation to allow more time for provider and patient interaction, preserving the patient-healthcare provider relationship [26].

*3.* *Equity and the Patient and Family Engagement*

The data on various healthcare outcomes clearly demonstrates the effect of structural racism and bias (both implicit and explicit) driving an international call to action towards more equitable healthcare ecosystems [27-29]. Health Opportunity and Equity (HOPE) initiative showed that the health status and outcomes, access to care, and socioeconomic status in the US vary by race and ethnic group [30]. The ongoing Covid-19 pandemic has highlighted gaps in accessibility and equity [29]. To close this gap of variation of the health outcomes, a global strategic framework with specific measures to focus on operationalizing equity and anti-racism as part of the healthcare providers’ everyday practices is required [28]. Applying the patient and family-centered care perspective, an example of the risk factor contributing to the inequity in the patient and family engagement, health outcomes, and patient safety is the language used for communication with the patients and the families. Karthik et al. described a pragmatic four-tier approach with measurable elements to overcome this issue, focusing on level 1) access to language interpreter; level 2) transition of care with similar interpreter services; level 3) measuring the quality of care for patients requiring interpreters; finally targeting level 4) socioeconomic and environmental factors that led to such language barriers [28]. Multiple organizations, including the Institute for Healthcare Improvement, have created conversation-ready toolkits and scripts in various languages to help healthcare providers conduct conversations with patients from different backgrounds [31,32]. To improve the overall quality of care and enhance patient and family engagement, it has become increasingly crucial for healthcare organizations and providers to morally invest in the strategies that help improve health equity [33]. The new models of care and payment for the healthcare services need to increasingly imbed the healthcare equity-based measures into their frameworks as the healthcare systems transform towards a better future.

4. Leverage Health Policy and Regulatory Frameworks for Rapid Adaptation of Patient and Family Engagement Strategies

The adoption of telehealth and removal of multiple medical licensures related hurdles through temporary declarations and waivers for out-of-state medical licenses of clinicians at the level of various US states and at the federal level by the Centers for Medicare and Medicaid Services early during the Covid-19 pandemic provides a prime example of the significant role that health policy and regulatory framework play to improve access to medical care and patient-centered care [19]. The whole concept of de-regulation and pan-national medical licensing has to be balanced with overarching safety for the protection of the citizens/public when credentialing. Health policy across the globe should continue its pivotal role in ensuring a framework to provide healthcare ‘a fundamental human right’ for all [33]. Continued support for telehealth incentives and a matching payment model by the health policymakers and regulators is vital to enhance shared-decision making, patient and family engagement, and genuine co-production to help patients live healthier lives [19,34,35]. Secondly, there is an urgent need to move the care out of the hospitals and closer to communities in home-based settings to avoid unnecessary emergency department visits and hospitalizations by providing acute care in the home or helping older adults safely “age in place” [34,35]. Thus, a combination of incentivization, deregulation, and additional forms of support is required, including reimbursement for home-based hospital services and removing barriers to safe and effective alternatives to the hospital for managing chronic diseases. Thirdly, the problems related to the social determinants of health that lead to poor health outcomes can be addressed by conducting more intersectoral work through community-based organizations that understand best what their communities need the most. By moving the money to education, job creation, criminal justice reform, immigration support systems, climate change, and increasing coordination with healthcare systems, we can increase health among populations and lower health care costs [33,34]. Finally, by integrating the health policy and regulatory frameworks with the patient-and-family-centered care frameworks, the legislators need to prioritize patient-and-family-centered outcome-based healthcare measurements and incentives. This type of approach will help ensure that value-based payment incentive systems will be paying for the real changes in health outcomes that patients and their families value the most [34,35].

## Conclusions

The healthcare systems across the globe have faced an unprecedented time over the course of last year marked by the Covid-19 pandemic. There is a stronger than ever call to action for healthcare organizations, leaders, and healthcare providers to use enhanced strategies to engage the patients and families and co-produce the healthcare services outcomes. An uphill task for the healthcare industry leaders is to harvest the value-adding practices from global healthcare systems, leading to speedy and equitable improvement of patient-and family-centered outcomes, lower healthcare delivery cost, and increase the value of care delivered to the patients.
